# Supplementary material for: Inverse J-Shaped Relationship of Dietary Carbohydrate Intake with Serum Klotho in NHANES 2007–2016
Source: Nutrients. 2023 Sep 13;15(18):3956. doi: 10.3390/nu15183956 (PMC10537068; doi:10.3390/nu15183956)
Supplement: Supplementary file 1 [file nutrients-15-03956-s001.zip › nutrients-2577533-supplementary.pdf]

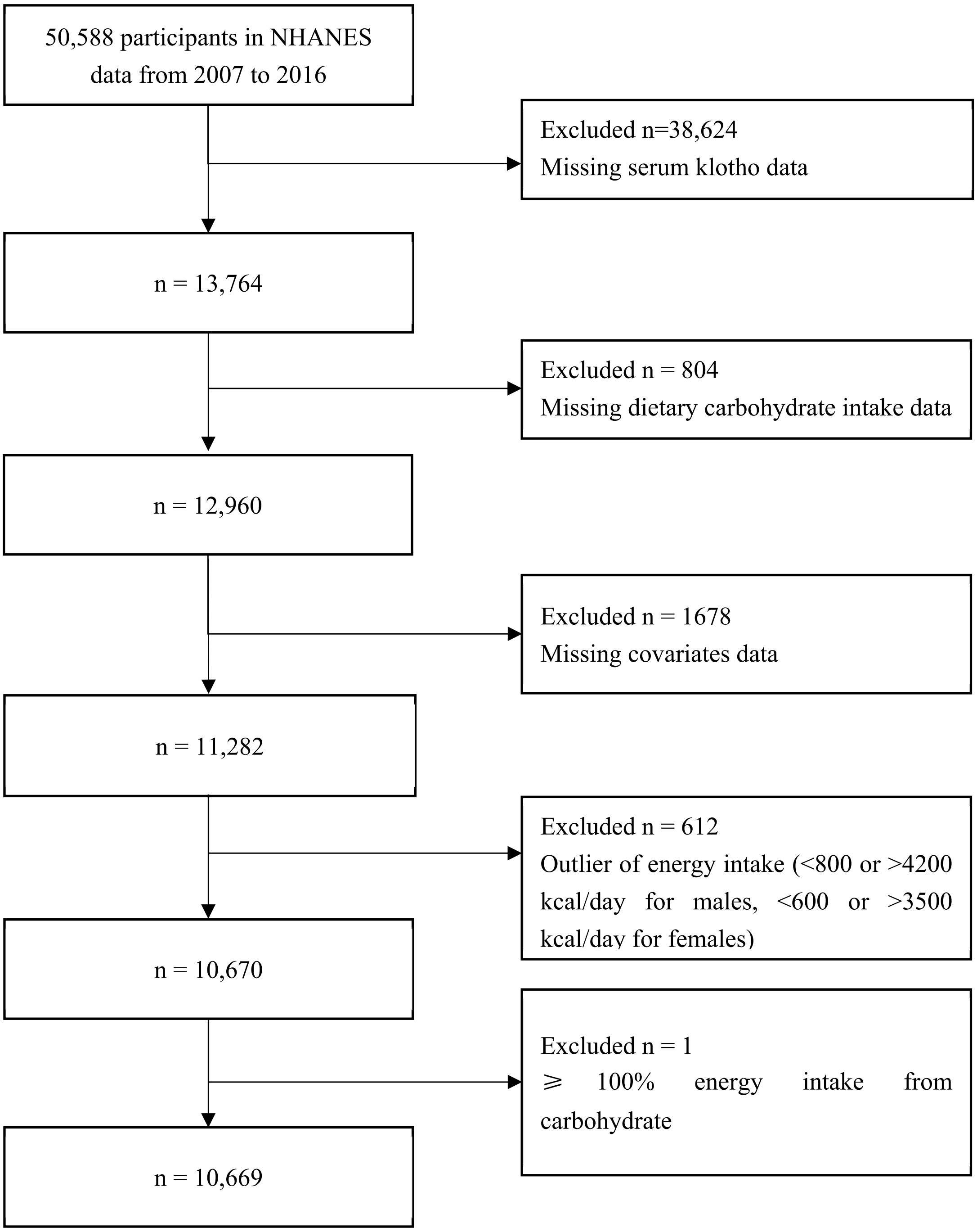

Figure S1. Flow chart of the study participants

Table S1. The association between the protein energy percentage and serum Klotho levels among all participants.

Participants	N	Models	Percent changes (%) and 95% CI	*p* Value
All	10,669	Crude model	2.20 (0.58, 3.85)	0.009
		Adjusted model*	2.49 (0.76, 4.26)	0.006

*Adjusted for age, gender, BMI, race, education level, PIR, serum cotinine, alcohol drinking, diabetes, hypertension, eGFR and dietary energy intake.

Abbreviations: CI, confidence interval.

Table S2. The association between the fat energy percentage and serum Klotho levels among all participants.

Fat intake, % of kcal	N	Crude model		Adjusted model*	
		Percent changes (%) and 95% CI	*p* Value	Percent changes (%) and 95% CI	*p* Value
Per 10% increases		0.43 (-0.49, 1.36)	0.363	0.70 (-0.24, 1.65)	0.149
Q1 (< 27.71)	2667	Ref	Ref	Ref	Ref
Q2 (27.71 to 33.74)	2667	3.08 (0.69, 5.52)	0.013	3.16 (0.87, 5.50)	0.009
Q3 (33.74 to 39.85)	2667	3.30 (0.56, 6.11)	0.020	1.63 (1.20, 6.75)	0.006
Q4 (≥39.85)	2668	2.32 (-0.10, 4.80)	0.064	3.48 (0.54, 5.43)	0.019
p for trend			0.106		0.035

*Adjusted for age, gender, BMI, race, education level, PIR, serum cotinine, alcohol drinking, diabetes, hypertension, eGFR and dietary energy intake.

Abbreviations: CI, confidence interval.

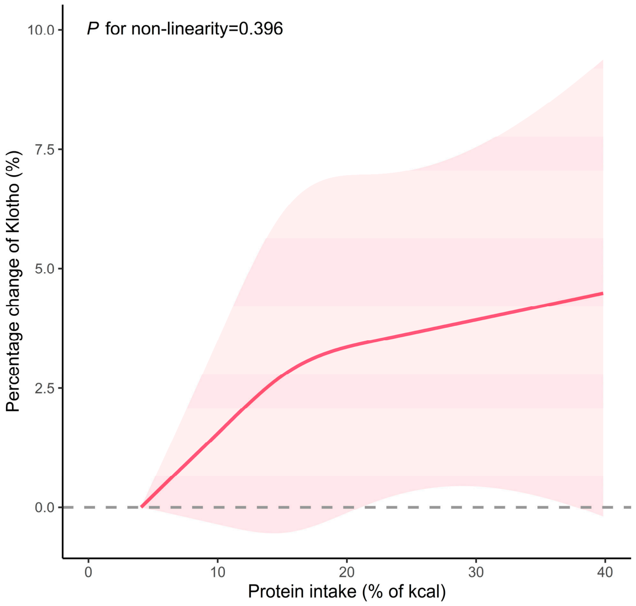

Figure S2. The dose-response relationship between the protein energy percentage and serum Klotho levels. The restricted cubic spline curve was carried out with 3 knots at the 10th, 50th, and 90th percentiles of fat energy percentage. The reference point was the maximum percentage change of Klotho. The solid line represented point estimation on the association of the protein energy percentage with serum Klotho, and the shaded portion represented 95% CI estimation. The model was adjusted for age, sex, BMI, PIR, education attainment, ethnicity, serum cotinine,

alcohol drinking, diabetes, hypertension, eGFR and dietary energy intake.

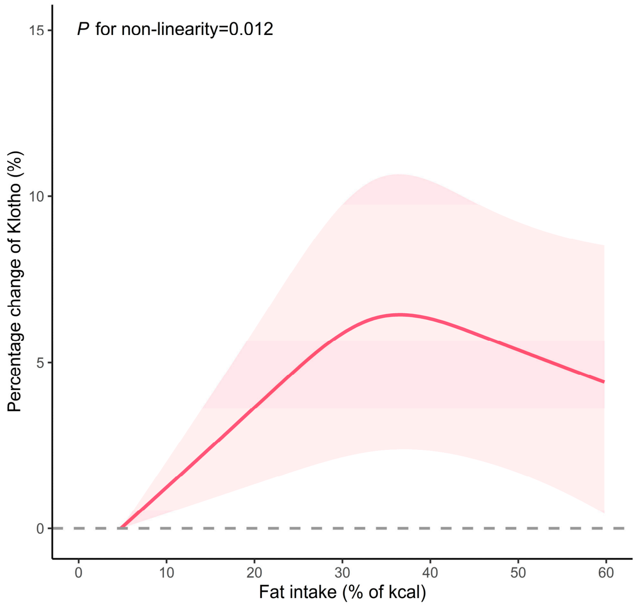

Figure S3. The dose-response relationship between the fat energy percentage and serum Klotho levels. The restricted cubic spline curve was carried out with 3 knots at the 10th, 50th, and 90th percentiles of fat energy percentage. The reference point was the maximum percentage change of Klotho. The solid line represented point estimation on the association of the fat energy percentage with serum Klotho, and the shaded portion represented 95% CI estimation. The model was adjusted for age, sex, BMI, PIR, education attainment, ethnicity, serum cotinine, alcohol drinking, diabetes, hypertension, eGFR and dietary energy intake.

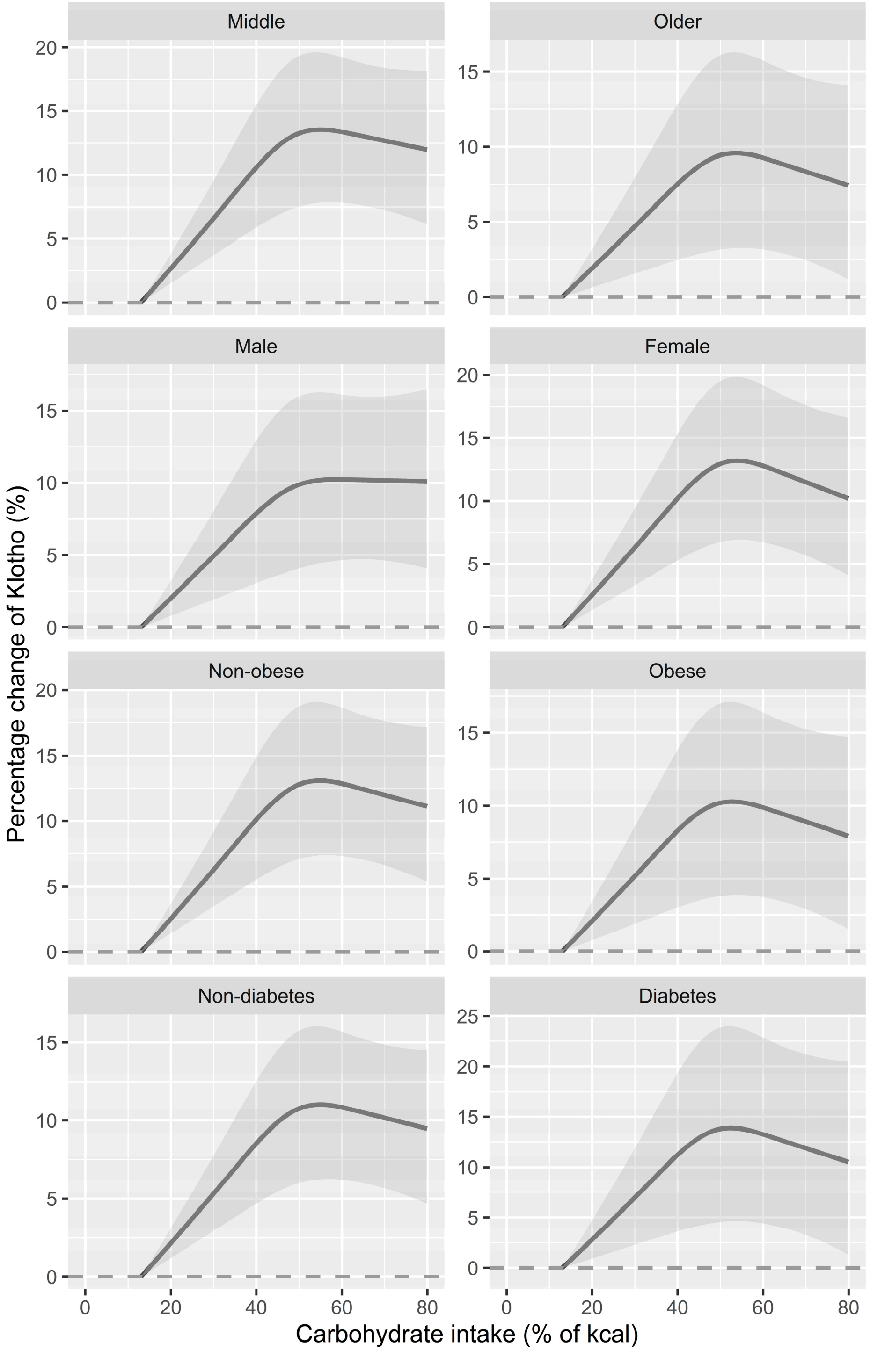

Figure S4. The dose-response relationship between the carbohydrate energy percentage and serum Klotho, stratified by age, sex, BMI and diabetes among 10,669 participants. The restricted cubic spline curve was carried out with 3 knots at the 10th, 50th, and 90th percentiles of carbohydrate energy percentage. The reference point was the maximum percentage change of Klotho. The solid line represented point estimation on the association of the carbohydrate energy percentage with serum Klotho, and the shaded portion represented 95% CI estimation. The model was adjusted for age, sex, BMI, PIR, education attainment, ethnicity, serum cotinine, alcohol drinking, diabetes, hypertension, eGFR, dietary energy intake, and dietary fiber.
